# Editorial: Insights in antimicrobials, resistance & chemotherapy: 2022

**DOI:** 10.3389/fmicb.2023.1310156

**Published:** 2023-10-19

**Authors:** Lucinda J. Bessa, Mona Shaaban, Rustam Aminov

**Affiliations:** ^1^Egas Moniz Center for Interdisciplinary Research (CiiEM), Egas Moniz School of Health and Science, Almada, Portugal; ^2^Department of Microbiology and Immunology, Faculty of Pharmacy, Mansoura University, Mansoura, Egypt; ^3^The School of Medicine, Medical Sciences, and Nutrition, University of Aberdeen, Aberdeen, United Kingdom

**Keywords:** novel antimicrobials, novel therapies, photodynamic therapy, antimicrobial resistance, efflux pumps, susceptibility tests, antimicrobial resistance genes, mobile genetic elements

Antimicrobial resistance (AMR) is a global pandemic that has to be comprehended and dealt with by a multidisciplinary approach, one of which is implemented in One Health initiative (Rayan, 2023). Within this framework, a multitude of research fields are advancing, including the development of innovative diagnostic tools, novel potential treatments/antimicrobials, improved epidemiological surveillance of specific pathogens and their AMR, and uncovering the mechanisms of AMR and their transmission. Thus, the main objective of this Research Topic was focused on gathering some recent developments in the areas listed above, with 15 contributions to the collection.

Development of novel antimicrobials and therapies remains a priority area in the age of ever-growing resistance of pathogens to the existing antimicrobials and other infection control measures. Identification, isolation and characterization of new antibiotic producers was a prevalent approach in the golden age of antibiotic discovery (Aminov, 2010). Since then, the rate of discovery of new classes of antibiotics from natural habitats has fallen dramatically. However, some ecological niches could still be under-explored for this purpose. Bioprospecting of Actinobacteria strains from mangrove rhizosphere by Ye et al. revealed antimicrobial, immunosuppressive and anticancer potential of these isolates. Another approach toward novel antimicrobials is a complete synthetic route. Menghani et al. investigated dithiocarbamate derivatives and some of them demonstrated a potent copper-dependent activity against pathogenic streptococci, including *in vivo* activities in a *Streptococcus pneumoniae* infection model.

Other potential avenues for control and management of bacterial infections could be physical approaches such as photodynamic therapy (PDT). It can be especially useful for treatment of bacterial infections, which form biofilms and thus notoriously resistant toward various antimicrobials (Lebeaux et al., 2014). Ong et al. compared the efficacy of antimicrobial blue light (aBL), levofloxacin and rifampin against *Staphylococcus aureus* ATCC 6538 in the planktonic and biofilm states. The efficacy of aBL might depend on *S. aureus* phenotype, and further research is necessary to determine whether it can be a plausible and applicable alternative to antimicrobial treatments. The antibacterial effect of PDT can be enhanced with the use of photosensitisers and Meng et al. investigated ornithine-porphyrin conjugates *in vitro* and *in vivo* for this purpose. Photosensitiser 4d demonstrated a high inactivation efficiency against multi-drug resistant *Proteus mirabilis* and accelerated wound healing via its bactericidal effect.

Nanoparticles display potent antimicrobial activities and considered to be one of the potential alternatives to antibiotics (Wang et al., 2017). Cell membrane-coated nanoparticles (CM-NPs) offer additional advantages serving as biomimetic nanoparticles, and Song et al. extensively reviewed potential therapeutic applications of CM-NPs against bacterial infections, including those forming biofilms. Besides, the use of CM-NPs can be combined with photodynamic, sonodynamic and photothermal therapies. Still, the use of CM-NPs faces several challenges from the preparation and application standpoints, and these have to be addressed before it could be considered as a viable therapeutic option.

Continuous updates on AMR mechanisms, especially against the drugs of last resort, remains a priority. Ding et al. reviewed the situation with resistance to one of these drugs, colistin. They concluded that, besides the plasmid-encoded *mcr* and chromosome-mediated lipopolysaccharide synthesis-related locus variations, efflux pumps could be also a factor contributing to colistin resistance.

Standard antimicrobial susceptibility tests performed in clinical microbiology laboratories are time-consuming and laborious. Thus, physicians frequently prescribe empirical therapies, often based on broad-spectrum antimicrobials (Gajic et al., 2022). Barth et al. evaluated a rapid susceptibility test to polymyxins, either from colonies grown on agar or directly from positive blood cultures, using MALDI-TOF based on the “direct on target microdroplets growth assay” (DOT-MGA), with some modifications. A total of 239 carbapenem-resistant clinical isolates of Enterobacterales and non-fermenting Gram-negative bacilli were tested for polymyxin resistance by DOT-MGA, in parallel with the standard broth microdilution method. A high concordance between the two tested methods, with more than 97% of general categorical agreement, was found, indicating that the adapted DOT-MGA can be a useful technique for rapid evaluation of susceptibility to polymyxins.

Another possibility to determine antimicrobial susceptibility could be through the analysis of bacterial genomes for the presence of the known AMR genes (Ellington et al., 2017). With the increasing affordability of whole genome sequencing, many clinical microbiology laboratories can implement this approach. Eriksen et al. performed a simple BLAST analysis to identify the nearest penicillin-binding protein (PBP)-profile of selected *S. pneumoniae* strains from Denmark and also international strains of non-*S. pneumoniae* Mitis-group streptococci (MGS). These genetic data were compared to the corresponding MIC phenotypes. The authors concluded that the genotypic susceptibility prediction was accurate for Danish *S. pneumoniae* isolates, particularly for those with the recognized PBP-profiles. However, susceptibility was poorly predictable for non-*S. pneumoniae* MGS.

Mobile genetic elements (MGEs), including plasmids, transposons, integrons, insertion sequences, gene cassettes and resistance islands play a dominant role in evolution and ecology of the microbial world (Aminov, 2011). In this role, MGEs serve as the main mechanisms of AMR acquisition by pathogenic bacteria (Algarni et al., 2022). Several articles in this Research Topic highlighted the role of MGEs in this process. Algarni et al. used computational-based approaches and reached an *in silico* prediction that AMR genes are diverse among plasmids replicon types, and multiple genes can be widely distributed across the plasmids circulating within enteric pathogens. Naderi et al. aimed to identify association between MGEs and aminoglycoside resistance in 315 *Acinetobacter baumannii* strains isolated from patients admitted to hospitals in Tehran, Iran. A total of 97 *A. baumannii* isolates belonged to the global clone 2, and they all harbored at least one MGE carrying aminoglycoside resistance genes, located either on the chromosome within the genomic resistance islands or on plasmids. Zhang et al. found that the *bla*_KPC − 2_- and *bla*_NDM − 1_ genes are co-harbored on an IncR plasmid, pCF2075-1, in three carbapenemase-producing *Citrobacter freundii* isolates, and the genes can be transferred through transposition. Ota et al. attempted to characterize *bla*_GES_-encoding plasmids from a single-hospital sewage sample in Japan. All 11 bacterial isolates with *bla*_GES_ (four *Enterobacter* spp., three *Klebsiella* spp., three *Aeromonas* spp., and one *Serratia* spp.) were classified as genetically distinct strains, and the corresponding *bla*_GES_-encoding plasmids were also diverse, belonging to the IncP-6, IncC, IncF and IncW incompatibility groups. At the same time, all *bla*_GES_ genes were located on the class 1 integron cassette of the Tn3 transposon-related region.

Continuous surveillance of bacterial pathogens and their AMR in humans and animals within One Health approach is crucial for understanding their epidemiology, and it serves as basis for clinical decision-making (Velazquez-Meza et al., 2022). In this Research Topic, Garrine et al. investigated antibiotic susceptibility and clonality of *S. aureus* strains isolated from blood cultures of children admitted to the Manhiça District Hospital in Mozambique during 2001–2019. AMR profiles of *S. aureus*, generated during the study, would allow a more targeted clinical management of pediatric patients with bacteremia in this region. In another Research Topic article, Morais et al. found a high frequency of methicillin resistance and a variety of clonal lineages with different AMR profiles among a collection of *Staphylococcus pseudintermedius* strains isolated from skin and soft-tissue infections in companion animals in Lisbon, Portugal. The data generated in this study would also help to select optimal antibiotic treatments, which may differ from the recommended guidelines.

Analysis of publication activity on certain pathogens via bibliometric analysis can give an idea how a particular field is developing and what could be the emerging areas of research in it (Ninkov et al., 2022). Yuan et al. conducted a bibliometric analysis of academic publications involving *Helicobacter pylori* during the past decade. This analysis can serve as a guide for the *H. pylori* research community to identify recent developments, research hotspots, and future trends in the area.

The main themes of the Research Topic “*Insights in antimicrobials, resistance, and chemotherapy: 2022*” covered the development of novel antimicrobials and treatments, contributed to better understanding of less known AMR mechanisms, introduced innovative antimicrobial susceptibility tests, uncovered the role of MGEs in AMR, and improved epidemiological surveillance of specific pathogens and their AMR. As editors of this topic, we anticipate this Research Topic would be useful to the research community. We are currently running another series of the topic, which is also dedicated to exploring novel developments, current challenges, recent discoveries, and future prospects within the field of antimicrobials, resistance and chemotherapy (https://www.frontiersin.org/research-topics/59624/insights-in-antimicrobials-resistance-and-chemotherapy-2023#overview). The themes of the topic include but not limited to: novel antimicrobials/treatments, AMR mechanisms, MGEs, and novel antimicrobial targets. We expect another successful topic that would provide the research community with some of the most recent updates in the area.

## Author contributions

LB: Writing—original draft, Writing—review and editing. MS: Writing—review and editing. RA: Writing—original draft, Writing—review and editing.
